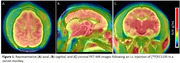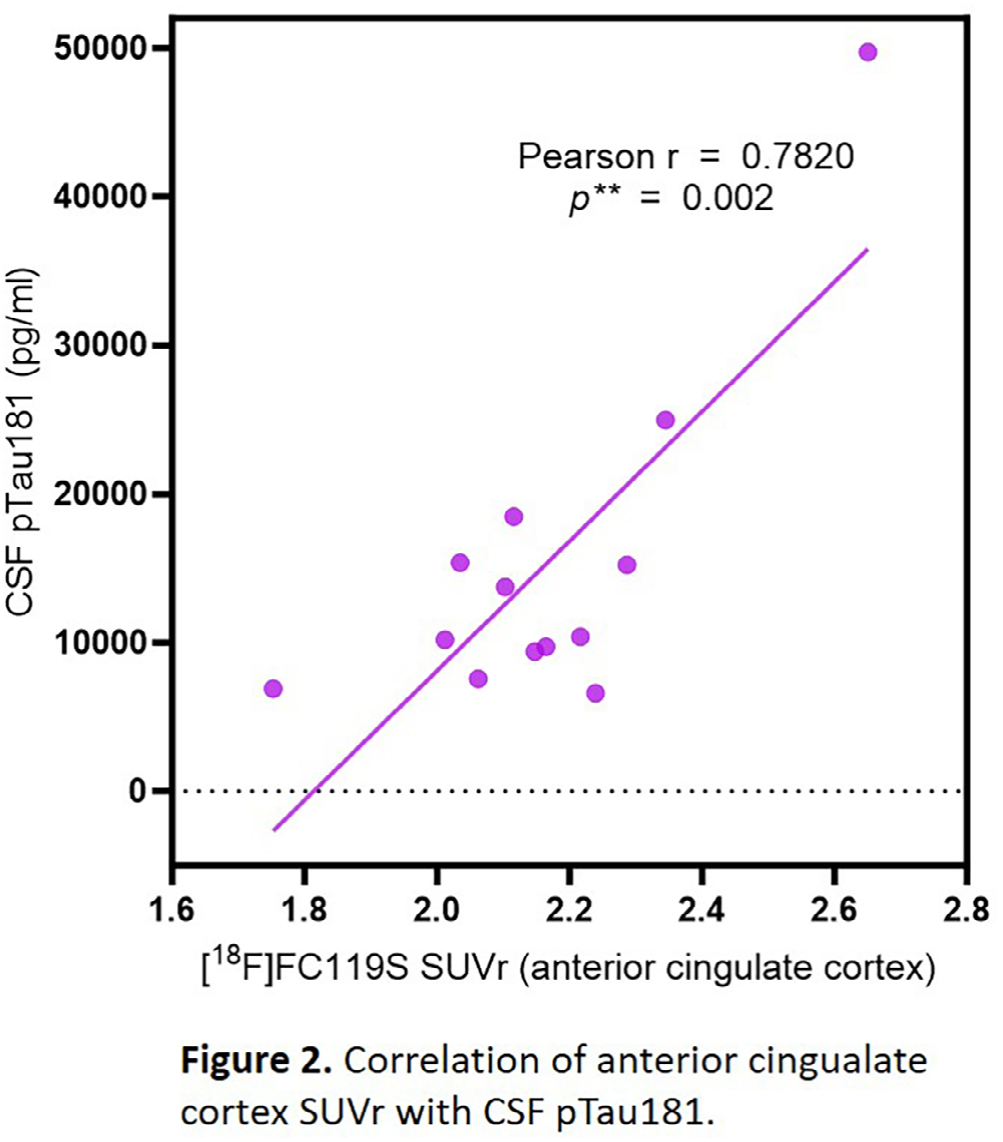# PET imaging utility of a novel Aβ‐tracking PET radiotracer, [^18^F]FC119S in aged vervet monkeys

**DOI:** 10.1002/alz.089843

**Published:** 2025-01-09

**Authors:** Bhuvanachandra Bhoopal, Brett M. Frye, Avinash Bansode, Mack Miller, Naresh Damuka, Krishna Kumar Gollepalli, Richard A. Barcus, Samuel N. Lockhart, Courtney L. Sutphen, Matthew J. Jorgensen, Suzanne Craft, Thomas C. Register, Christopher T Whitlow, Carol A. Shively, Kiran K. Solingapuram Sai

**Affiliations:** ^1^ Wake Forest University School of Medicine, Winston‐Salem, NC USA

## Abstract

**Background:**

Older vervet monkeys are an excellent model for studying age‐associated Aβ deposition; however, they have high proportions of low‐affinity Aβ sites compared to human brains. Commonly used Aβ PET radiotracers are most useful in detecting high affinity Aβ fibrils. Measuring real‐time levels of low affinity Aβ fibrils through PET provides critical information of early AD progression. The radiotracer [^18^F]FC119S has high binding strength for Aβ_1–42_ protein aggregates (0.16 nM) and AD brain homogenates (13–15 nM). A pilot clinical trial showed significantly higher hippocampal and cortical uptake in AD patients over cognitively normal participants. Here we report the fully‐automated [^18^F]FC119S radiochemistry and preliminary imaging and fluid biomarker correlation data in aged vervet monkeys.

**Methods:**

Radiochemistry followed [^18^F]^¯^‐assisted nucleophilic substitution of mesityl precursor and deprotection of Boc group. [^18^F]FC119S(∼0.37GBq)‐based brain PET (0‐120min) and T1‐weighted whole‐brain MRI scans were conducted in aged vervets (19‐27 y, n=14). CSF and plasma Aβ_40_, Aβ_42_, pTau181, and NfL were measured in samples taken close to the time of PET. Standard Uptake Values with cerebellum as reference (SUVr) and time‐activity‐curves (TACs) were calculated from PET/MR (Figure 1). SUVrs of anterior and posterior cingulate, hippocampus, frontal, parietal, temporal, and occipital lobes were correlated with age, MRI outcomes (grey and white matter volumes), and fluid biomarker data.

**Results:**

[^18^F]FC119S production was highly reproducible (n>25 runs) in high (>97%) radiochemical purities, and molar activities (∼140 GBq/µmol). TACs showed favorable washout kinetics from all brain regions. SUVrs of hippocampus and cortical parietal lobe positively correlated with age and SUVrs of all the regions analyzed negatively correlated with MRI deep gray and white matter volumes. Among the high SUVr regions, cortical anterior cingulate SUVr was highly positively correlated (Figure 2) with CSF pTau181 (r=0.78, **p=0.002).

**Conclusion:**

Preliminary [^18^F]FC119S PET evaluations agrees with human PET data in vervets with high uptake in regions associated with Aβ deposition. Among all fluid biomarkers analyzed, CSF pTau181 was the best correlate of all the brain PET SUVrs. Association of Aβ PET imaging data with synaptic density and microtubule data in the same cohort is being evaluated to explore the potential neurodegeneration pathways in aged vervet monkeys.